# Carotenoids moderate the effectiveness of a Bt gene against the European corn borer, *Ostrinia nubilalis*

**DOI:** 10.1371/journal.pone.0199317

**Published:** 2018-07-10

**Authors:** Daniela Zanga, Georgina Sanahuja, Matilde Eizaguirre, Ramon Albajes, Paul Christou, Teresa Capell, Paul Fraser, Chris Gerrisch, Carmen López

**Affiliations:** 1 Department of Crop and Forest Sciences, University of Lleida-Agrotecnio Center, Lleida, Spain; 2 School of Biological Sciences, Royal Holloway, University of London, Egham, Surrey, United Kingdom; University of Texas at Austin, UNITED STATES

## Abstract

We assessed the effectiveness of a biofortified maize line (4BtxHC) which accumulates high levels of antioxidant carotenoids that also expressed the insecticidal Cry1Ac *Bacillus thuringiensis* (Bt) gene against the European corn borer *Ostrinia nubilalis*. This line had been previously engineered to accumulate carotenoids specifically in the seed endosperm, whereas the Bt gene was expressed constitutively. The concentrations of Bt toxin (Cry 1Ac) in the leaves of the 4Bt and 4BtxHC lines were not significantly different at 47±6 μg/g of fresh weight (FW); neither were they in the kernels of both lines (35±3 μg/g FW). The kernels and leaves were toxic to the larvae of *O*. *nubilalis*. However, the insecticidal activity was substantially lower (ca. 20%) than that of lines that expressed only Bt in spite that the two lines showed a quantity of toxin not significantly different in kernels or in leaves. Although the reduced effectiveness of Cry1Ac in kernels may not be entirely surprising, the observation of the same phenomenon in vegetative tissues was unexpected. When semi-artificial diets containing kernels from 4Bt supplemented with different levels of β-carotene were used in insect bioassays, the β-carotene moderated the effectiveness of the Bt similarly to the plant material with carotenoid enrichment. To elucidate the biochemical basis of the reduced effectiveness of Bt toxin in the carotenoid-enriched plants, we measured the activity of three enzymes known to be implicated in the detoxification defence, namely, catalase, superoxide dismutase and glutathione S-transferase. Whereas Cry1Ac expression significantly increased SOD and CAT enzymatic activity in the absence of carotenoids, carotenoids, either in 4BtxHC or in artificial diets enriched with β-carotene, significantly lowered CAT activity. Carotenoids can therefore moderate the susceptibility of the maize borer *O*. *nubilalis* to Cry1Ac, and we hypothesize that their role as antioxidants could explain this phenomenon via their scavenging of reactive oxygen species produced during Cry1Ac detoxification in the larvae. The involvement of this mechanism in the decreased mortality caused by Cry1Ac when carotenoids are present in the diet is discussed.

## Introduction

Bt crops engineered to express the insecticidal Cry protein(s) of the entomopathogenic bacterium *Bacillus thuringiensis* (Bt) have been grown commercially since 1996. In 2016, 185.1 million ha of Bt crops were planted, most of them with two or more Bt toxins or stacked with herbicide tolerance [1]. Whereas most commercialized biotech crops exhibit enhanced agronomic traits such as insect resistance and/or herbicide tolerance, many new biofortified crops with enhanced nutritional traits are just under development or nearing commercialization. Metabolic engineering is used to produce one or more nutritionally important molecules such as vitamins, antioxidants or health-promoting fatty acids. A maize line accumulating high levels of β-carotene and other nutritionally important carotenoids in the endosperm, Carolight®, has been described [2] and tested in the field for its agronomic performance [3].

Little is known of the effects of biofortified crops on herbivorous insects, and few studies have been devoted to known how carotenoid enhancement in host plants may affect herbivore survival, development, and behaviour. Carotenoids are responsible for visual pigments and body colour in insects and crustaceans, have strong antioxidant activity and act as insect hormone precursors, but their transition along food webs has not been elucidated [4]. The oxidative cleavage of carotenoid substrates produces volatile apocarotenoids, which are important compounds in herbivore-plant communication, since they can be either strongly attractive or repellent to insects [5]. For example, Carolight® was found to be less attractive to the maize stem borer *Sesamia nonagrioides* for egg deposition [6]. It is important to investigate how plants with enhanced nutritional traits such as increased carotenoids behave in terms of susceptibility to feeding, as herbivores may change their susceptibility to pest control agents on this basis.

Most commercial maize grown in Spain is derived from the MON810 transformation event and expresses the Cry1Ab toxin. Unlike Bt maize, however, Carolight® is susceptible to lepidopteran pests. Thus, c*ry1Ac* was engineered into Carolight®, and the resulting plants (referred to as 4BtxHC) were evaluated in a series of experiments to assess potential interference between the engineered nutritional and insecticidal traits, that is, if the insecticidal efficacy of the Bt trait may be affected by the carotenoids in the host plant. Little is known concerning the potential antagonistic or synergistic effects of carotenoids on the expression and effectiveness of Bt genes or the susceptibility of insects to Bt toxins. 4BtxHC expresses the carotenogenic genes maize phytoene synthase 1 (*Zmpsy1)* and *Pantoea ananatis* phytoene desaturase (*PacrtI*) under the control of endosperm-specific promoters (wheat LMW glutenin and barley D-hordein promoters, respectively), while *cry1Ac* expression is controlled by the maize ubiquitin 1 constitutive promoter (Ubi-1 promoter). It is therefore possible that an interaction between the engineered gene products might occur, particularly in the 4BtxHC endosperm, where all these genes are expressed. Carotenoids and Cry1Ac might have a synergistic or antagonistic interaction once inside the larval body, due to the ability of carotenoids to scavenge reactive oxygen species (ROS), which are implicated in detoxification pathways in the insect. ROS are normally produced during cellular metabolism. However, environmental stresses, such as chilling, drought, salinity, and pathogen attack, lead to excessive ROS production due to disruption of cellular homeostasis [7]. The increased production of ROS during environmental stresses can be very harmful to cells by causing lipid peroxidation, protein oxidation, nucleic acid damage, enzyme inhibition, and activation of programmed cell death pathways, ultimately leading to cell death [7]. An effective antioxidant system, comprising both non-enzymatic and enzymatic antioxidants, is needed to scavenge excess ROS [8]. Enzymatic antioxidants include superoxide dismutase (SOD), catalase (CAT) and glutathione S-transferase (GST) [8]. Ascorbate (AsA), glutathione (GSH), carotenoids, tocopherols, and phenolics serve as powerful non-enzymatic antioxidants within the cell. SOD is responsible for the catalytic dismutation of the potentially toxic superoxide anion radical to hydrogen peroxide [9]. CAT is present in peroxisomes and catalyses the decomposition of hydrogen peroxide to yield oxygen and water [10]. GST catalyses the conjugation of the reduced form of GSH to xenobiotic substrates for detoxification. Various studies have reported modified activities of many enzymes of the antioxidant defence system in plants to combat the oxidative stress induced by various environmental stresses [7].

We therefore tested the hypothesis that the accumulation of high levels of carotenoids might interfere with the effectiveness of Bt toxins by scavenging ROS produced during Bt toxin detoxification processes in lepidopteran insect pests that feed on maize. We used the European corn borer *Ostrinia nubilalis*, which is one of the main targets of all Bt commercial maize varieties grown in Europe and the USA. We measured the activities of CAT, SOD, and GST, which are likely involved in the detoxifying processes of *O*. *nubilalis*, and we assessed how the addition of β-carotene to artificial diets with or without Cry1Ac affected larval survival and the activity of the above mentioned enzymes.

## Material and methods

### Plant material

Maize (*Zea mays*) M37W and the transgenic lines 4Bt, expressing *Bacillus thuringiensis cry1Ac* in the M37W genetic background, and DKC6667YG (Dekalb®, containing the MON810 event), expressing Bt Cry1Ab, were germinated in the greenhouse of the University of Lleida. The line 4Bt was generated by introducing the *Cry1Ac* gene into M37W by direct transformation and bringing this transformant to homozygosity for subsequent experiments including crossing with HC (which has M37W as background) to generate 4BtxHC [11].

Plants were grown at 28/20°C day/night temperature with a 10 h photoperiod and 60–90% relative humidity for the first 50 days, followed by maintenance at 21/18°C day/night temperature with a 16-h photoperiod. M37W seeds were obtained from CSIR (Pretoria, South Africa). 4Bt seeds were available in our laboratory [11]. An elite event with the highest Bt protein accumulation was identified and self-pollinated to obtain a 4Bt homozygous line.

By crossing 4Bt with Carolight® (HC), an engineered high-carotenoid maize expressing maize *psy1* and *Pantoea ananatiscrtI* in the M37W genetic background [2], a new line was obtained: 4BtxHC. A homozygous 4BtxHC line was obtained through cycles of self-pollination.

### Quantification of Cry1Ac toxin content in plant material

The content of Cry1Ac protein in plant material was determined before the start of the experiments using the Agdia Bt-Cry1Ab/Cry1Ac ELISA kit (Agdia Inc., Elkhart, Indiana, USA). For calibration, Cry1Ac standards at 8.0,6.0, 4.0, 2.0, 1.0, 0.5 and 0.25 ng/ml were used, and measurements were made with a VICTOR^3^ Multilabel Plate Counter (PerkinElmer Life and Analytical Science, Madrid, Spain) following the procedure described in [12].

### Quantification of beta-caroten in kernel-based diets

The β-carotene content in the diet was measured by HPLC. Extraction, HPLC separation, photodiode array detection and quantification of carotenoids have been described in detail previously [13].

### Insect rearing

Larvae of *O*. *nubilalis* were obtained from the culture maintained in our entomology laboratory, which is renewed every three or four generations with insects collected in field areas in Lleida with no Bt maize cultivation. Larvae were maintained at 25°C and high humidity (>60%) under a 16:8 h light:dark photoperiod and reared on a semi-artificial diet according to Eizaguirre and Albajes [14].

### Bioassays

Four sets of bioassays were performed to test the toxicity of plant material (leaves or kernels) to *O*. *nubilalis* larvae or to measure enzyme activity in the larvae.

#### Efficacy of Cry 1Ac against *O*. *nubilalis* (experiment 1)

To test the efficacy of the Cry1Ac toxin, larval mortality was assessed using 4Bt plants (expressing Cry1Ac), a positive control [commercial plants (DKC6667Y, Dekalb®) based on event MON810 (Cry1Ab)], and a negative control (near-isogenic wild-type plants, M37W). Four independent plants were used as biological replicates for each of the three tested lines. Leaves from 40-day-old plants were washed in water and quickly blotted, and 4–5 cm sections were placed in a container (diam. 5.5 cm x 3 cm height) lined with moist filter paper and containing insect larvae. Each replicate consisted of ten containers, and five newly hatched larvae reared in the laboratory were released into each container; therefore, in each replicate, 50 insects were tested. The containers were maintained at 25°C and high humidity (>60%) under a 16:8 h light:dark photoperiod. Insect mortality was recorded after five days of exposure.

#### Bt toxicity in 4Bt vs 4BtxHC plants (experiment 2)

This experiment was designed to compare the mortality caused by Cry 1Ac in larvae fed on 4Bt and 4BtxHC plants. Two different diets were used for this experiment; one consisted of leaves and the other kernels. Two additional non-Bt control plants, HC and M37W, were also used as negative controls in the experiment with leaves and kernel. In addition to the mortality, in tests performed with leaves, leaf consumption at the end of the experiment was also measured, and in the kernel diet experiment, larval development duration from the beginning of the experiment to pupation, pupation success, and pupal weight were measured. The leaf tests followed the same protocol as in Experiment 1, but L2 larvae instead of neonate larvae were used. The tests performed with diets containing kernels of the different plant materials also used L2 larvae, and they consisted of adding a 0.5 * 0.5 * 0.5 cm^3^ cube of diet [14] to each container and replacing it with fresh diet every 48/72 h. The mortality of the L2 larvae fed on leaves was recorded after five days of exposure, and in the kernel diet test, mortality was recorded weekly until the end of week 4.

#### Effects of added dietary β-carotene on Bt toxicity (experiment 3)

To test for potential interference between β-carotene and Cry1Ac, we added a beta-carotene amount similar to the one contained in HC and 4BtxHC (4Bt+0.6) and we chose 4Bt+6 and 4Bt+60 to test if higher beta-carotene concentrations could further increase the survival of larvae exposed to Bt toxin. Mortality in larvae fed on a diet containing kernels of 4Bt plus different quantities (0.6, 6.0, and 60 mg/100 g of diet, referred to as 4Bt+0.6, 4Bt+6, and 4Bt+60, respectively) of β-carotene (TCI America-TCI Chemicals, CAS: 7235-40-7) was recorded weekly from L5 to pupation, until week 3 when all larvae fed the non-Bt diet had pupated. A diet with kernels of M37W (the same diet as in the experiment 2 was used) plus different amounts of β-carotene was used as a non-Bt control, and a diet with kernels of BtxHC with no added β-carotene was used as a negative control. The rest of the conditions in this experiment were as in previously described bioassays performed with kernel diets.

#### Effects of carotenoids on detoxification enzyme activity in *O*. *nubilalis* (experiment 4)

The activities of the CAT, SOD and GST enzymes involved in the detoxification processes in the insect midgut were measured in two separate experiments in which *O*. *nubilalis* larvae were fed on different diets: in the first experiment (Experiment 4a), the treatments were M37W, HC, Bt and BtxHC diets made with kernels (the same diet as mentioned in experiment 2 was used) while in the second experiment (Experiment 4b), different quantities of β-carotene (0.6, 6, and 60 mg/100 g of diet) were added to 4Bt diets (Bt, Bt0.6, Bt6, Bt60) and compared to the enzyme activity in insects fed on the wild type (M37W) and M37W+6 (the intermediate quantity of β-carotene added to 4Bt). A number of larvae comprised between 7 and 11 individually reared were used at the L5 stage for each treatment. Each larva was fed with a cube of semi-artificial diet for 2 days and then immediately frozen in liquid nitrogen to be used for enzyme activity analyses. To determine SOD and CAT activity, entire larval body samples were homogenized by sonication in PBS-Tween (0.5 ml PBS-Tween per 100 mg of tissue), followed by centrifugation at 10,000 × g for 15 min at 4°C. Supernatants were used for determinations according to the kit manufacturer’s instructions. The sensitivity of the assay for SOD was 0.044 U/mL (K028-H1; Arbor Assays, Ann Arbor, MI, USA); for CAT, it was 0.052 U/mL (K033-H1; Arbor Assays). U/mL is units of SOD/Catalase activity per ml, normalized for protein concentration. Protein levels were measured using the Bradford method, based on the principle of protein-dye binding [15]. For GST activity, entire larval body samples were homogenized by sonication in 110 μl of HEPES buffer 0.05 M (pH 7), followed by centrifugation at 10,000 × g for 15 min at 4°C. Supernatants were collected, and 30 μl of each sample was pipetted in duplicate in a black ELISA plate. To each well, 170 μl of the following mix was added: 3 mM GSH (cofactor), 0.3 mM monochlorobimane and 50 mM HEPES buffer, pH 7. The plate was incubated at room temperature for 20 minutes in the dark. Fluorescence was measured at 380 nm excitation and 465 nm emission with a VICTOR^3^ Multilabel Plate Counter. Values were expressed as units of fluorescence per mg of protein per minute.

### Statistical analysis

The influence of the different plant lines on the measured variables (larval mortality, leaf consumption, non-lethal effects, and enzyme activity) was analysed by one-way (plant line) ANOVA. For all comparisons, the level of P≤0.05 was considered significant. Percentages were transformed by ASIN (SQRT (%/100)) to normalize the data. Means were compared when needed by least significant difference (LSD). All statistical analyses were done using JMP® statistical package [16].

## Results

### Levels of Bt toxin in leaves and kernels

The concentration of Bt toxin in the leaves of DKC6667Y (Cry1Ab) was 39±1 (mean ± S.E.) μg/g fresh weight (FW), whereas the concentrations in the leaves of the 4Bt and 4BtxHC lines (Cry1Ac) were not significantly different at 47±6 μg/g FW. The Cry1Ac concentration in kernels (35±3 μg/g FW) was not significantly different between 4Bt and 4BtxHC, similar to the levels in the leaves.

### β-Carotene content in kernel-based diets

β-Carotene was not present in the M37W and 4Bt diets; in the 4Bt+60 diet, β-carotene accumulated at 15.8 ± 0.16 μg/g dry weight (DW), whereas the HC, 4BtxHC and 4Bt+0.6 diets had similar β-carotene contents (1.09 ±0.06 μg/g DW). β-Carotene was also present at similar levels in the M37W+6 and 4Bt+6 diets (3.02 ± 0.17 μg/g DW).

### Efficacy of Cry 1Ac against *O*. *nubilalis* (experiment 1, Fig 1)

This experiment aimed to test the efficacy of the Cry1Ac toxin by comparing larval mortality when using 4Bt plants (expressing Cry1Ac), with a positive control [commercial plants (DKC6667Y, Dekalb®) based on event MON810 (Cry1Ab)], and with a negative control (near-isogenic wild-type plants, M37W).

**Fig 1:**
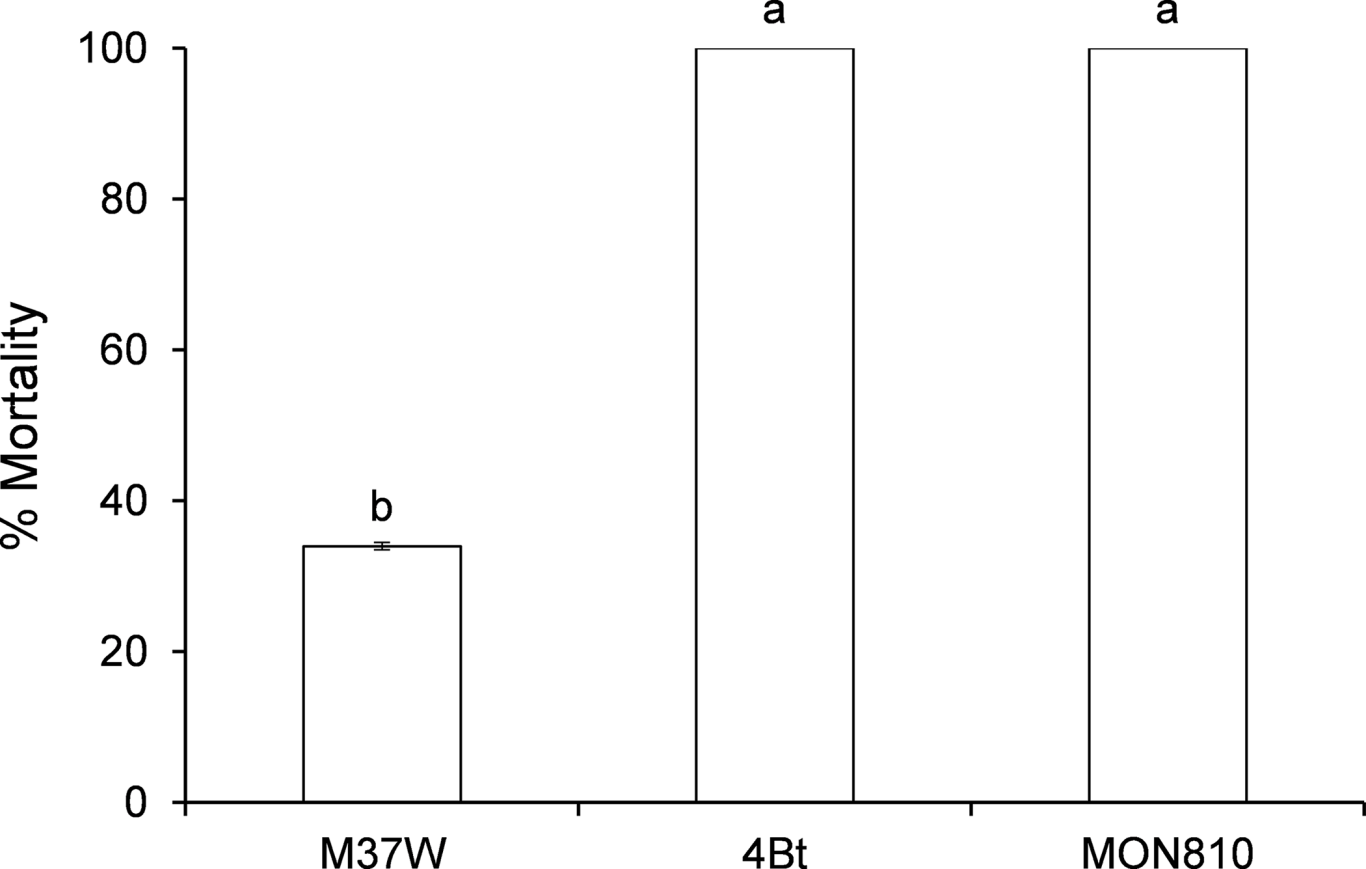
Mortality (mean±S.E.) of *Ostrinia nubilalis* neonate larvae when fed on leaves of different maize lines; larval mortality was recorded after five-day exposure (experiment 1). [F = 65.88; P<0,001; df = 2,27]. 4Bt contained the Cry1Ac toxin, MON810 contained the Cry1Ab toxin, and M37W was the non-Bt near isogenic line of 4Bt. Different letters indicate statistically significant differences between the varieties (P< 0.001) when data were analysed by one-way (plant line) ANOVA.

*O*. *nubilalis* larvae feeding on 4Bt leaves exhibited 100% mortality after five days of exposure (Fig 1). This mortality was not significantly different to that caused by the commercial variety DKC6667YG (MON810 containing Cry1Ab) (F = 65.88; P<0.001; df = 2,27), a variety sown in Spain for many years that is resistant to *O*. *nubilalis* in the field. Mortality in the non-Bt M37W (near-isogenic line of 4Bt) variety was within the range of natural mortality of neonate larvae in the field.

### Toxicity of Bt and BtxHC to *O*. *nubilalis* (experiment 2, Figs 2–4)

Experiment 2 was designed to compare the mortality caused by Cry 1Ac in larvae fed on 4Bt and 4BtxHC plants when two different diets were used, one consisted of leaves and the other kernels.

**Fig 2:**
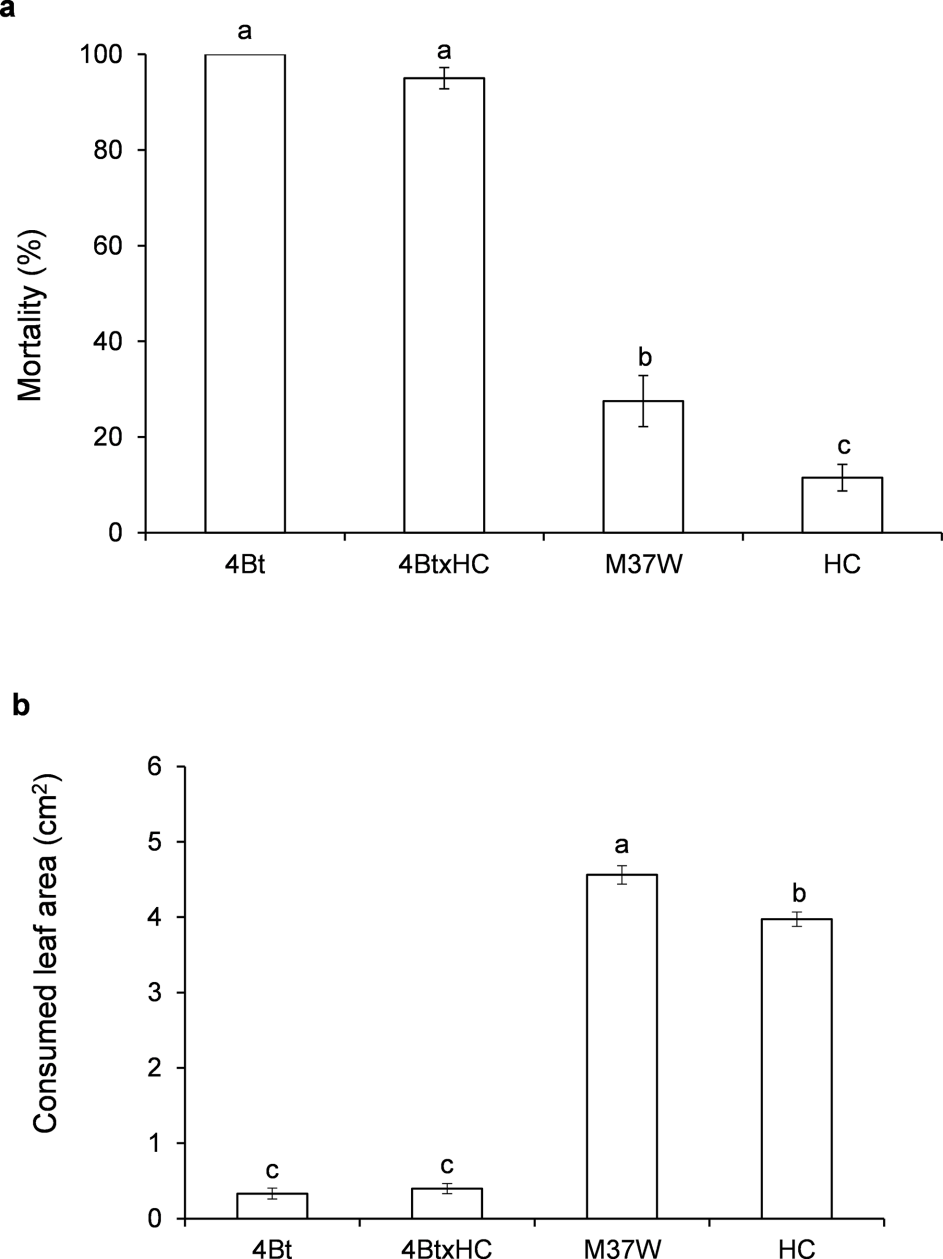
(a) Mortality (mean±S.E.) of second instar *Ostrinia nubilalis* larvae fed on leaves of different maize lines; larval mortality was recorded after five-day exposure [F = 186.74; P<0.001; df = 3,156]. (b) Area (mean±S.E.) of leaf consumed by larvae in Fig 2(A) [F = 581.97; P<0.001; df = 3,23]. 4Bt contained the Cry1Ac toxin, HC was the transformed line with high carotenoid content, M37W was the near isogenic line, and 4BtxHC was the line with the two traits. Different letters indicate statistically significant differences between the lines (P< 0.001) when data were analysed by one-way (plant line) ANOVA.

When non-Bt plants were compared, mortality on HC was significantly lower (11.5%) than that on M37W (27.5%), whereas there were no significant differences between the two Bt lines (Fig 2A) (F = 187.74; P<0.001; df = 3,156). No significant differences were found in the amount of leaf consumed by the larvae when they were fed on the two Bt lines (4Bt or 4BtxHC) (Fig 2B), whereas in addition to the lower mortality on the leaf, consumption by larvae fed on HC was significantly lower than consumption by larvae fed on the near-isogenic line M37W (F = 581.97; P<0.001; df = 3,23).

**Fig 3:**
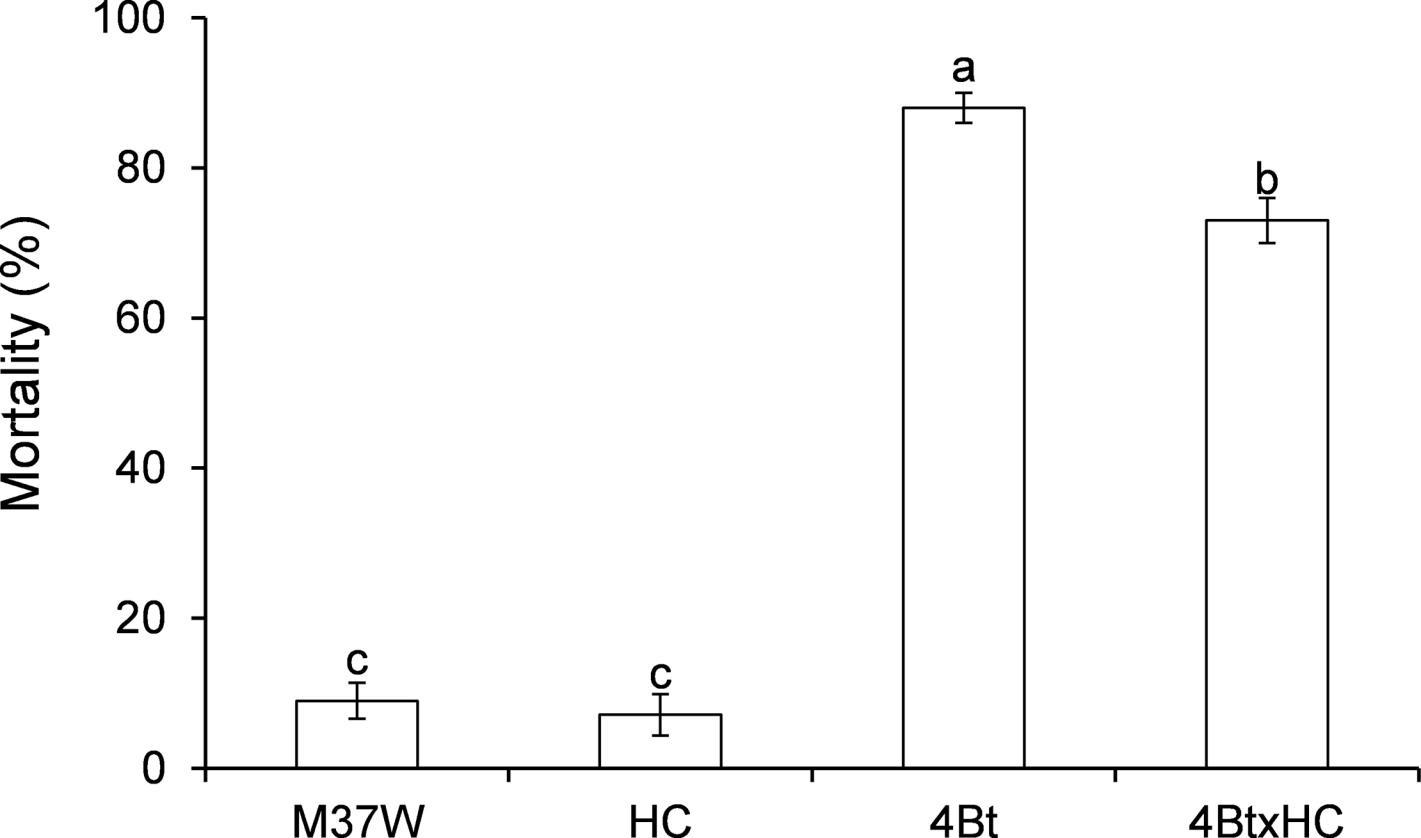
Mortality (mean±S.E.) of second instar *Ostrinia nubilalis* larvae fed on a diet made with kernels of different maize lines; larval mortality was recorded after four week-exposure [F = 186.74; P<0.001; df = 3,156]. 4Bt contained the Cry1Ac toxin, HC was the transformed line with high carotenoid content, M37W was the near isogenic line, and 4BtxHC was the line with the two traits. Different letters indicate statistically significant differences between the lines (P< 0.001) when data were analysed by one-way (plant line) ANOVA.

In the bioassay with a diet containing maize kernels, after 4 weeks of exposure, *O*. *nubilalis* larvae exhibited significantly (20%) higher mortality on 4Bt than on 4BtxHC diets (Fig 3) (F = 131.42; P<0.001; df = 3,108).

**Fig 4:**
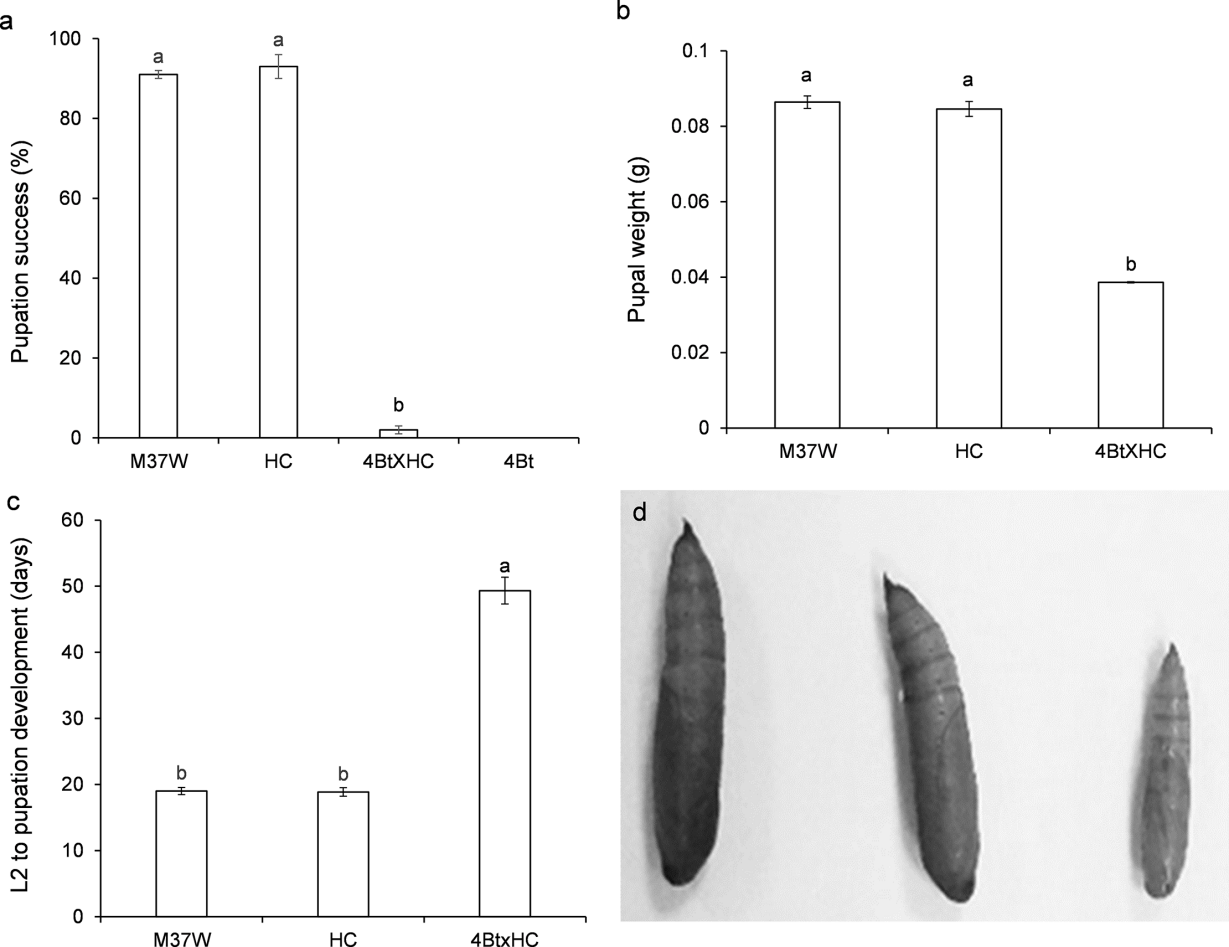
Non-lethal effects (mean±S.E.) on *Ostrinia nubilalis* larvae fed from second instar to pupation on a diet made with kernels of different maize lines. 4Bt contained the Cry1Ac toxin, HC was the transformed line with high carotenoid content, M37W was the near isogenic line, and 4BtxHC was the line with the two traits. **a)** Percentage of pupation [F = 541.27; P<0.001; df = 3,156]. **b)** pupal weight [F = 34.04; P<0.001; df = 2,56]. (g) **c)** Larval development from bioassay initiation to pupation [F = 98.16; P<0.001; df = 2,56]. and **d)** Pupal size of *O*. *nubilalis* larvae fed for four weeks on a diet made with grains of M37W (control, left figure), HC (middle), and 4BtxHC (right figure) plants. Different letters indicate statistically significant differences between the lines (P< 0.001) when data were analysed by one-way (plant line) ANOVA.

All the larvae fed on the 4Bt line either died without pupating or remained as larvae without pupating; a few (2%) of those fed on 4BtxHC pupated within the 4-week period. Larvae fed on the non-Bt lines (M37W and HC) showed no differences from each other in pupation success (92%) (Fig 4A) (F = 541.27; P<0.001; df = 2,81). In the latter, pupal weight was significantly decreased (60% less) (Fig 4B) and size was smaller (Fig 4D) than that of non-*Bt*-fed larvae (F = 34.04; P<0.001; df = 2,56). Larvae of 4BtxHC developed significantly slower than those developed on of non-Bt lines (F = 98.16; P<0.001; df = 2,56) (Fig 4C).

### Addition of β-carotene to diets and effects on Bt toxicity to *O*. *nubilalis* (experiment 3, Fig 5)

To test for potential interference between β-carotene and Cry1Ac, mortality in larvae fed on a diet containing kernels of 4Bt plus different quantities of β-carotene was recorded weekly from L5 to pupation.

**Fig 5:**
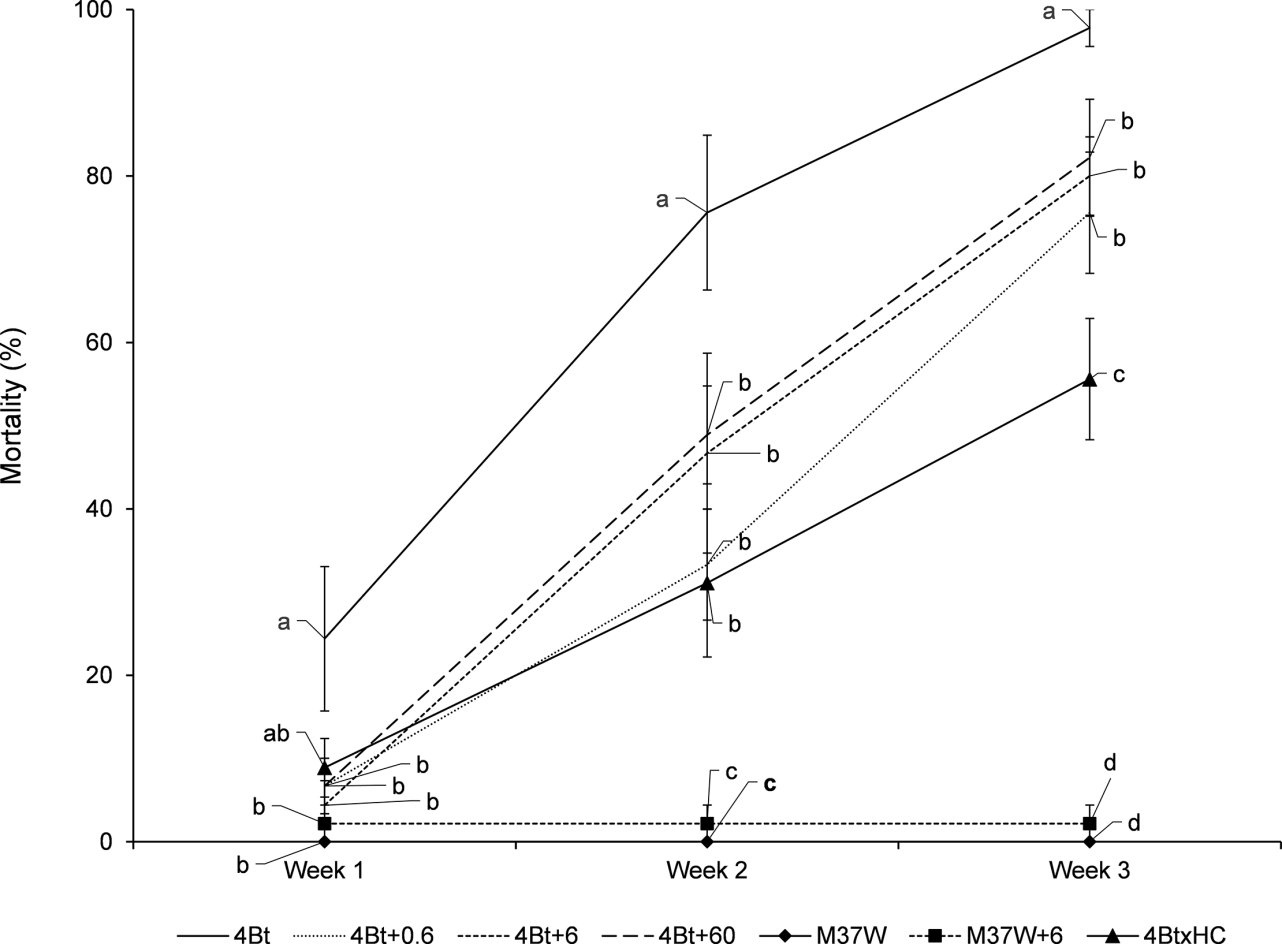
Changes in mortality percentage (mean±S.E.) of *Ostrinia nubilalis* larvae fed on diets made with kernels of different maize lines when different quantities of β-carotene were added to the diet (in mg/100g diet). 4Bt contained the Cry1Ac toxin, M37W was the near isogenic line, and 4BtxHC was the line with the two traits. Fifth stage larvae were treated and mortality was recorded every week for three weeks. Week 1: [F = 2.68; P<0.02; df = 6,56]. Week 2: [F = 14.94; P<0.001; df = 6,56]. Week 3: [F = 56.25; P<0.001; df = 6,56]. Different letters indicate statistically significant differences between diets (P < 0.001) when data were analysed by one-way (plant line) ANOVA.

Addition of β-carotene to the diet made with 4Bt lowered larval mortality (20–35%) independently of the quantity of β-carotene added (Fig 5) (see statistical results of each recording week in the Fig 5). As in Experiment 2, mortality in larvae fed on a 4BtxHC kernel diet was significantly (up to 60%) lower than that of larvae fed on 4Bt (Fig 5). Two weeks after the beginning of the experiment, the mortality of the larvae fed on 4Bt plus β-carotene was not significantly different to that of larvae fed on 4BtxHC; however, at the end of the experiment, the effect of adding β-carotene to 4Bt did not reduce larval mortality (mortalities between 75.6% and 82.2%) as much as 4BtxHC did (mortality 55.6%). All larvae fed on non-Bt diets reached pupation, while no larvae fed on 4Bt or 4Bt+ β-carotene diets could pupate, and only a few individuals (4%) in the 4BtxHC group did. In these latter individuals, pupal weight was significantly lower than that of non-Bt-fed larvae (330±80 mg *vs* 870±150 mg).

### Effect of carotenoids and addition of β-carotene to Bt diet on CAT, SOD, GST enzyme activities in *O*. *nubilalis* (experiment 4, Fig 6A and 6B)

This experiment aimed to know whether the activities of the CAT, SOD and GST enzymes involved in the detoxification processes in the insect midgut could be affected when *O*. *nubilalis* larvae were fed on the 4 lines tested (M37W, HC, Bt, and BtxHC) or when different quantities of β-carotene were added to 4Bt diets in comparison with the enzyme activity in insects fed on the wild type (M37W) and M37W6.

**Fig 6:**
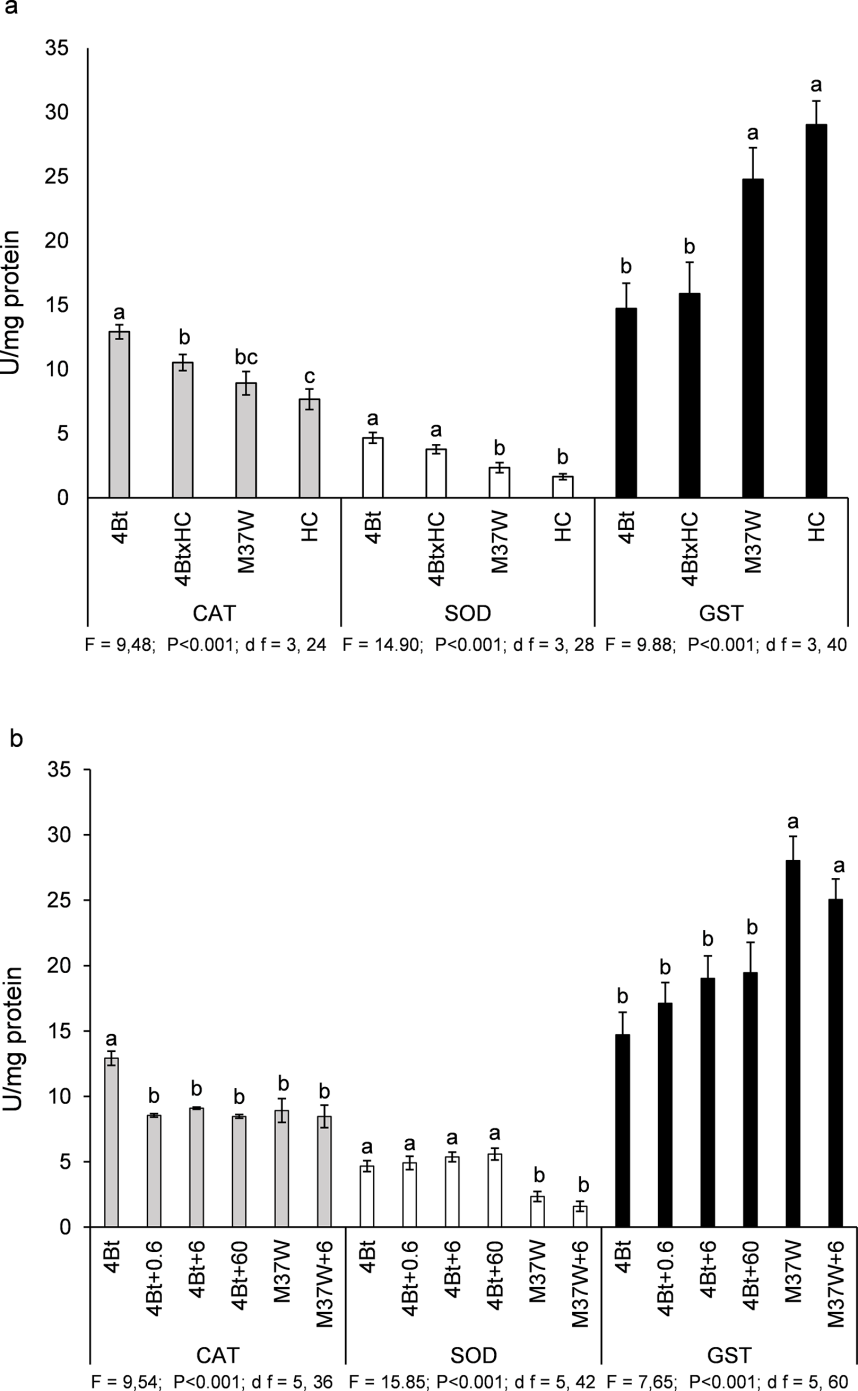
Effect (mean±S.E.) of Cry1Ac and carotenoids on CAT, SOD, GST activity on *Ostrinia nubilalis* larvae. Enzyme activity in the fifth stage larvae fed on **a)** diets made with grain of different maize lines (4Bt, HC, M37W, and 4BtxHC) **b)** diets made with different amounts of added β-carotene. In (a) the treatments were M37W, HC, 4Bt and 4BtxHC diets, while in (b), different quantities of β-carotene (0.6, 6, and 60 mg/100 mg of diet) were added to 4Bt and M37W diets (4Bt, 4Bt+0.6, 4Bt+6, 4Bt+60, M37W, M37W+6). Fifth stage larvae were used and results are shown two days after exposure. CAT: Catalase; SOD: Superoxide dismutase; GST: Glutathione S-transferase. Different letters indicate statistically significant differences between diets (P < 0.001) when data were analysed by one-way (plant line) ANOVA. Results of statistics shown in each of the figures.

Larvae fed on the 4BtxHC diet had significantly lower CAT activity than those fed on 4Bt, consistent with the lower larval mortality caused by the carotene-enriched Bt line (Fig 6A) (F = 9.48; P<0.001; df = 3,24). However, the differences between 4BtxHC and 4Bt were not significant in the case of SOD and GST enzymatic activities. As expected, the enzymatic activity in larvae fed on non-Bt diets was significantly different to that in larvae fed on 4Bt. The larvae fed on 4Bt showed 36% and 59% higher enzymatic activities of CAT (statistics shown above) and SOD (F = 14.90; P<0,001; df = 3,28), respectively, and 82% lower GST activity (F = 9.88; P<0.001; df = 3.40) than larvae fed on non-Bt diet did (average of M37W and HC) (Fig 6A). The addition of β-carotene to the diet made with 4Bt kernels caused a similar effect on enzyme activity. β-carotene significantly reduced the activity of CAT (F = 9.54; P<0.001; df = 5,36), 33% as average of all β-carotene treatments, but it did not significantly modify that of SOD or GST; the reduction in CAT activity was independent of the amount of β-carotene added (Fig 6B).

All data needed to draw each figure in this study are available as supporting information (S1 File)

## Discussion

Maize engineered with the Bt toxin Cry1Ac (4Bt) accumulated similar amounts of Bt toxin to plants from a commercial event (MON810) engineered with the very similar Cry1Ab Bt toxin. Both lines were comparably lethal to neonate larvae of the maize stalk borer *O*. *nubilalis* (Fig 1). The Cry1Ac toxin is structurally similar to Cry1Ab, which is efficient in controlling different lepidopteran maize pests, such as the borers *O*. *nubilalis*, *O*. *furnacalis* and *S*. *nonagrioides* [17,18, 19, 20, 21]. Through our transgenic 4Bt plants, we have shown that maize plants expressing *cry1Ac*, with Cry1Ac toxin at a concentration of 50 μg/g FW, are as effective as maize plants expressing *cry1Ab* in controlling young larvae of *O*. *nubilalis*. This result is consistent with those of previous studies reporting that Cry1Ab and Cry1Ac recognize with high affinity the same binding sites in the *O*. *nubilalis* midgut [22, 23].

The high-carotenoid content trait (HC) caused several effects on *O*. *nubilalis* larvae when introduced into M37W alone or in combination with the Cry1Ac trait (4BtxHC) (Fig 2). By itself, high carotenoid content caused a reduction in insect mortality and leaf consumption when larvae were fed on leaves compared with those with no carotenoid enhancement (M37W). When combined with the Bt trait, high carotenoid content caused lowered mortality due to the Bt toxin when the insects were fed on a diet made with the kernels of 4BtxHC engineered plants.

Carotenoids are substrates for carotene cleavage dioxygenases (CCD), which catalyse the cleavage of carotenoids to apocarotenoids [24] and have recently been demonstrated to have antixenotic effects in different herbivore species [5], particularly in the corn borer *S*. *nonagrioides* [6]. Reduction of mortality due to Bt by high carotenoid content was only found when larvae were fed on kernels (Fig 3) rather than leaves (Fig 2). This result is not unexpected, as the transgenic carotenoid pathway in 4BtxHC was engineered specifically in the endosperm. As the levels of the Cry1Ac toxin were very similar in 4Bt and 4BtxHC, other factors linked to the antioxidant role of carotenoids and detoxification processes could explain the differences in mortality.

Nutrition is recognized as an important factor that affects the susceptibility of insects to Bt toxins [25, 26]. Even a change in protein-to-carbohydrate ratio in the insect diet can lead to substantial differences in Bt toxin susceptibility, as reported by Deans et al [26], who measured a 100-fold increase in Cry1Ac LC_50_ for *Helicoverpa zea* larvae when they compared optimal diets (balanced P:C) *vs* carbohydrate-enriched diets. We attributed the reduced susceptibility of *O*. *nubilalis* to *Bt* toxins in our experiments to the enhanced antioxidant content in the carotenoid-enriched diets. Antagonistic effects between antioxidants and Bt toxins have been reported in a number of lepidopteran insects. Broderick et al [27] reported a dose-dependent extension of larval survival when they exposed larvae of gypsy moth (*Lymantria dispar*) to Bt toxin combined with GSH compared to Bt toxin alone [27]. In another recent study, a similar antagonism between the flavonoid quercetin and Cry1Ac was observed in *H*. *armigera* larvae [28]. The expected effect of Cry1Ac on pupation success and pupal weight was moderated when quercetin was introduced at various concentrations into the diet together with the Cry1Ac toxin, similar to our results when we compared pupation success in larvae fed on 4Bt and 4BtxHC. A similar effect was obtained when β-carotene was added to the diet made with 4Bt kernels. Addition of different quantities of β-carotene also lowered the toxicity of Cry1Ac to larvae fed on this diet but to a lesser extent than 4BtxHC did.

The decreased susceptibility to Cry1Ac in the presence of dietary carotenoids could be explained by the role of carotenoids as antioxidants in scavenging ROS produced during Cry1Ac detoxification processes in the larvae. This hypothesis is consistent with the increased CAT values found here in larvae fed on 4Bt in comparison with those fed on 4BtxHC or in larvae fed on 4Bt plus different amounts of β-carotene. Similarly, in *O*. *nubilalis*, Yao et al [29] reported an overexpression of one CAT gene after feeding with Cry1Ab toxin. Furthermore, in another lepidopteran species, *Spodoptera litura* higher CAT activity was measured when larvae were fed on kernels of two Bt maize hybrids expressing Cry1Ab [30]. Since many carotenoids can directly scavenge O2^–^ in the cell [31], carotenoids can exhibit competitive relations with CAT, neutralizing the H_2_O_2_ generated by O2^–^ dismutation. Furthermore, ^1^O_2_ scavenging also decreases the generation of O2^–^ [32]. Therefore, enhanced carotenoid content in tissues should be accompanied by a decrease in CAT activity in larvae fed on Bt, consistent with our results in this work, where CAT activity values were lowered by 4BtxHC compared to 4Bt. Note that high carotenoid content per se did not modify enzyme activity, as M37W and HC showed enzyme activities that were not significantly different. The fact that the addition of β-carotene to the diet made with 4Bt was as efficient as high carotenoid content in reducing CAT activity suggests that β-carotene is at least partially responsible for the overall effect of carotenoids. The same trend, although without statistically significant differences, was observed in the activities of two other enzymes commonly involved in detoxification processes in insects, SOD and GST, which were modified, although not significantly, in 4BtxHC in comparison with 4Bt.

As noted by Wang et al [33] and references therein, overexpression of GST has been associated with insecticide intake in insects, but recent studies have also shown that GST expression is reduced in Cry3Aa-intoxicated *Tenebrio molitor* larvae and in Cry1Ab resistant *O*. *furnacalis*. In our study, such a reduction in GST activity in Bt-fed larvae was also found in other studies [29, 34, 35]. Yao et al [29] who reported in *O*. *nubilalis* the downregulation of the GST gene after feeding with Cry1Ab toxin; Zhou et al [34] also observed that Cry1Ac reduced GST enzyme activity in *O*. *furnacalis*; and Gui et al [35] in another lepidopteran herbivore, *Bombyx mori*, observed that the ingestion of an entomopathogenic virus caused a reduction in GST activity.

We provide evidence that enhanced carotenoid content moderates the susceptibility of the corn borer *O*. *nubilalis* to Bt toxin. This protective effect was also observed when insects were fed on kernel diets with Cry1Ac supplemented with β-carotene, independently of the amount of β-carotene added. Furthermore, we hypothesized that the antioxidant activity of carotenoids, which decreases oxidative stress and protects cells from tissue damage by scavenging ROS, is at least one of the mechanisms underlying the antagonistic interaction between Cry1Ac and carotenoids. At least in part, the lower susceptibility of larvae to Cry1Ac when they are fed carotenoid-enriched diets is due to higher activity of CATs, one of the enzyme classes responsible for toxin detoxification. Our work brings to light important considerations in the design, deployment implementation and management strategies of biofortified crops, particularly when these crops also include Bt toxins and measures to prevent Bt-resistance development must be implemented.

## Supporting information

S1 File The file contains data set needed to draw Figs 1–6 on the effects of the exposure of *Ostrinia nubilalis* larvae to different treatments. (XLSX)
